# Diagnostic Value of Plasma Annexin A2 in Early-Stage High-Grade Serous Ovarian Cancer

**DOI:** 10.3390/diagnostics11010069

**Published:** 2021-01-04

**Authors:** Noor A. Lokman, Carmela Ricciardelli, Andrew N. Stephens, Thomas W. Jobling, Peter Hoffmann, Martin K. Oehler

**Affiliations:** 1 Discipline of Obstetrics and Gynaecology, Adelaide Medical School, Robinson Research Institute, The University of Adelaide, Adelaide, SA 5005, Australia; noor.lokman@adelaide.edu.au (N.A.L.); carmela.ricciardelli@adelaide.edu.au (C.R.); 2 Future Industries Institute, University of South Australia, Adelaide, SA 5001, Australia; peter.hoffmann@unisa.edu.au; 3 Centre for Cancer Research, Hudson Institute of Medical Research, Clayton, VIC 3168, Australia; andrew.n.stephens@hudson.org.au; 4 Department of Gynaecological Oncology, Monash Health, Clayton, VIC 3168, Australia; tjobling@bigpond.net.au; 5 Department of Gynaecological Oncology, Royal Adelaide Hospital, Adelaide, SA 5005, Australia

**Keywords:** annexin A2, plasma, ovarian cancer, CA125, early detection, biomarkers, HGSOC

## Abstract

Ovarian cancer (OC) is commonly diagnosed at advanced stage when prognosis is poor. Consequently, there is an urgent clinical need to identify novel biomarkers for early detection to improve survival. We examined the diagnostic value of the calcium phospholipid binding protein annexin A2 (ANXA2), which plays an important role in OC metastasis. Annexin A2 plasma levels in patients with high grade serous OC (*n* = 105), benign ovarian lesions (*n* = 55) and healthy controls (*n* = 143) were measured by ELISA. Annexin A2 levels were found to be significantly increased in patients with stage I (*p* < 0.0001) and stage IA (*p* = 0.0027) OC when compared to healthy controls. In the logistic regression models followed by receiver operating characteristics (ROC) curve analyses, plasma annexin A2 showed 46.7% sensitivity at 99.6% specificity in distinguishing stage IA OC patients from healthy controls and 75% sensitivity at 65.5% specificity in the diagnosis of stage IA versus benign ovarian tumors. In the diagnosis of stage IA OC versus normal controls, the combination of plasma annexin A2 and CA125 showed 80% sensitivity at 99.6% specificity (AUC = 0.970) which was significantly higher than for CA125 (53.3% sensitivity at 99.6% specificity; AUC = 0.891) alone. The diagnostic accuracy in distinguishing stage IA OC from benign ovarian disease when combining annexin A2 and CA125 (71.4% accuracy at 100% sensitivity) was almost twice as high compared to CA125 (37.1% accuracy at 100% sensitivity) alone. In conclusion, annexin A2 in combination with CA125 has potential as a biomarker for the early detection of OC and to predict malignancy in patients with ovarian lesions, warranting further investigations.

## 1. Introduction

Ovarian cancer (OC) is the gynecological malignancy with the highest mortality and accounts for an estimated 152,000 deaths worldwide each year [1]. The high mortality of OC is caused by the asymptomatic nature of early disease, resulting in over 70% of patients being diagnosed at advanced stage when the cancer has metastasized (International Federation of Gynecology and Obstetrics (FIGO) stage III and IV). Prognosis at this stage is poor and 5-year survival is only about 30%. In contrast, early OC, i.e., when it is still confined to the ovary (FIGO stage I), is associated with a 5-year survival rate of over 90% and is largely curable [2,3]. Therefore, early detection is the most effective way of improving OC survival. However, currently no effective early detection tests are available and population screening is therefore not possible.

Cancer antigen 125 (CA125), is the current gold standard protein biomarker in OC. Its utility, however, is limited to distinguishing benign from malignant ovarian lesions and to assess tumor load during treatment and follow up [4]. CA125 is only elevated in less than 50% of early stage OC patients and can be increased in patients with benign ovarian disease [5,6]. Consequently, CA125 on its own is not a reliable and sensitive tumor marker for early-stage OC detection.

Numerous efforts have been undertaken to develop a biomarker-based early detection test for OC (reviewed in [7]). Other circulating markers (such as human epididymis protein 4 (HE4), CA72.4, lysophosphatidic acid (LPA), transthyretin, prostasin, osteopontin, bikunin, vascular endothelial growth factor (VEGF) and many more) have been reported, but none of those markers showed sufficient sensitivity and specificity for OC screening [8,9,10]. In order to improve the diagnostic accuracy, multivariate index assays (e.g., risk of ovarian malignancy algorithm (ROMA) and OVA-1) were developed, but clinical diagnostic utility remained limited and restricted to treatment monitoring and surveillance [11,12,13].

Annexin A2 (ANXA2) is a calcium phospholipid binding protein that is present on the surface of various tumor cells [14,15]. We recently reported an important role of annexin A2 in high grade serous ovarian cancers (HGSOC) metastasis [16] and for prognosis [17]. However, thus far, no studies have investigated the potential value of plasma annexin A2 in the diagnosis of OC. In this study, we assessed the diagnostic performance of plasma annexin A2, either alone or in combination with CA125 in patients with OC, benign ovarian tumors and healthy controls.

## 2. Materials and Methods

### 2.1. Clinical Samples

Blood samples were collected with approval from the Research Ethics Committee at the Royal Adelaide Hospital, Adelaide, South Australia (Protocol number: 140101) and written patient consent. Additional samples of patients with HGSOC (stage I-IV), benign ovarian lesions and healthy controls were obtained from the Hudson Institute of Medical Research (Clayton, Australia), Ontario Tumor Bank (Toronto, ON, Canada) and Precision Med Inc. (Solana Beach, CA, USA). Blood samples of early-stage breast cancer patients were sourced from Conversant Biosciences Inc. (Huntsville, AL, USA). The clinicopathological characteristics of the patient cohort are detailed in Supplementary Tables S1 and S2.

Plasma EDTA samples were collected into vacutainer blood collection tubes (Greiner Bio-One, Kremsmünster, Austria), centrifuged at 3000 rpm for 10 min at room temperature and stored at −80 °C until assayed. Plasma was chosen as a sample type in this study, as we found higher annexin A2 levels in serum when compared to matching plasma EDTA samples (Supplementary Figure S4a). There was a nonsignificant correlation between serum annexin A2 and plasma annexin A2 (Supplementary Figure S4b, Correlation coefficient = 0.364, *p* = 0.245).

### 2.2. Annexin A2 Measurements

Annexin A2 levels were measured using a commercial human annexin A2 ELISA kit as per manufacturer’s instructions (USCN Life Science Inc., Wuhan, China). Briefly, plasma samples were diluted 1:10 in PBS (pH 7.4) and 100 μL of standards or samples added into each well in duplicates and incubated for 2 h at 37 °C. Subsequently, detection reagent A (100 μL) was added and incubated for 1 h followed by detection reagent B (100 μL) for 30 min. The substrate solution (90 μL) was added into each well for 10 min followed by stop solution (50 μL). The absorbance was measured at 450 nm using the Triad series multimode detector (Dynex Technologies, Chantilly, VA, USA). The detection limit of the assay was 0.321 ng/mL and the intra assay and inter assay coefficient of variation (CV) was 18% and 26%, respectively.

### 2.3. CA125 Measurements

CA125 levels were measured with the Siemens Advia Centaur XP automated analyzer at the Institute of Medical Veterinary Science (IMVS) (SA Pathology, Adelaide, Australia).

### 2.4. Statistical Analysis

Statistical analyses were performed using SPSS for Windows (Version 26.0, SPSS Inc., Chicago, IL, USA) and GraphPad Prism for Windows (Version 8.0, La Jolla, CA, USA). The measurements for plasma annexin A2 and CA125 were log (natural) transformed due to skewness in the logistic regression model. The predictive value probabilities for either annexin A2 or CA125 alone and combined plasma annexin A2 + CA125 were obtained to create the ROC curve and acquire AUC values, sensitivity and specificity. The proportion of true positives (TP), true negatives (TN), false positives (FP) and false negatives (FN) from the logistic regression model was calculated using the SAS software for Windows (SAS Institute, Inc, Cary, NC, USA). The accuracy is the percentage of correctly classified cases and was calculated from (TP + TN)/(TP + TN + FP + FN). The statistical significance between the diagnostic groups was determined by Kruskal–Wallis test and Dunn’s multiple comparison test. Spearman’s rho correlation test was used to determine the correlation between CA125, annexin A2 and age. Statistical significance was accepted at *p* < 0.05.

## 3. Results

### 3.1. Plasma Annexin A2 Levels Are Elevated in Stage I Ovarian Cancer

**Figure 1:**
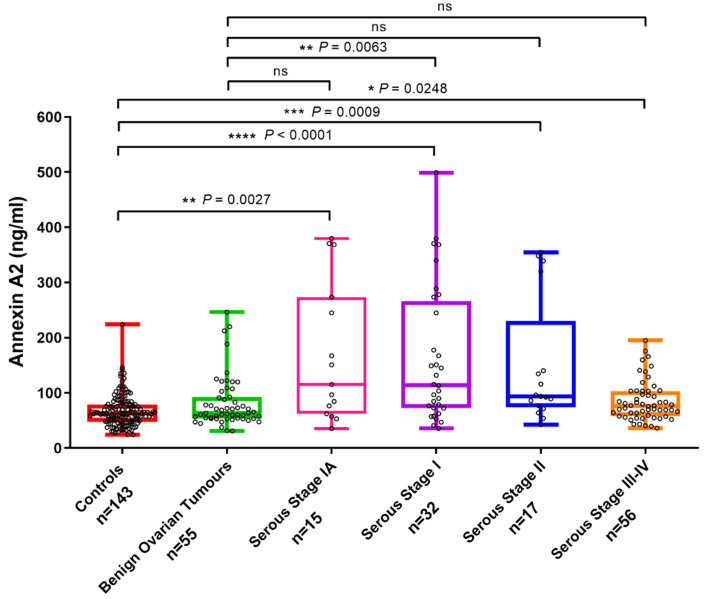
Plasma annexin A2 levels in patients with OC, benign ovarian tumors and healthy controls. Box and whisker plots representing annexin A2 levels measured in stage IA (*n* = 15), stage IA-IC (*n* = 32), stage II (*n* = 17) and stage III-IV (*n* = 56) OC, benign ovarian tumors (*n* = 55) and healthy controls (*n* = 143). Median values for plasma annexin A2: stage IA (115.3 ng/mL, range: 35.7–379.7), stage IA-IC (114.2 ng/mL, range: 35.7–499.1), stage II (93.6 ng/mL, range: 42.6–354.7), stage III-IV (76.2 ng/mL, range: 36.1–194.9) OC, benign ovarian tumors (63.5 ng/mL, range: 31.1–246.1) and healthy controls (62.7 ng/mL, range: 24.2–224.3). Comparison between patient groups was performed by Kruskal–Wallis and Dunn’s multiple comparison tests. (ns = not significant, * *p* value < 0.05, ** *p* value < 0.01, *** *p* value < 0.001, **** *p* value < 0.0001).

Plasma annexin A2 levels were significantly elevated in patients with FIGO stage I (1A-IC) (2.42-fold increase, *p* < 0.0001) and IA (2.51-fold increase, *p* = 0.0027) OC compared to healthy controls. Significantly increased plasma annexin A2 levels were also found in patients with stage II (2.2-fold increase, *p* = 0.0009) and stage III/IV (1.26-fold increase, *p* = 0.0248) OC versus healthy controls (Figure 1). Plasma annexin A2 levels were significantly higher in patients with stage I (1A-IC) cancers versus patients with benign ovarian tumors (2-fold increase, *p* = 0.0063). However, no significant difference was observed between plasma annexin A2 in patients with stage IA (2.07-fold increase, *p* = 0.13), stage II (1.81-fold increase, *p* = 0.08) or stage III/IV OC (1.04-fold increase, *p* = 0.10) versus benign ovarian lesions.

We observed a weak correlation between plasma annexin A2 and CA125 in stage I OC and healthy controls (Supplementary Figure S1, Correlation coefficient = 0.154, *p* = 0.042). Annexin A2 levels in stage I-IV OC patients and healthy controls showed no correlation with patients’ age (Supplementary Figure S2, Correlation coefficient = 0.040, *p* = 0.53). Additionally, we also assessed plasma annexin A2 in early-stage breast cancers (stage I and II) and no significant difference was observed compared to healthy controls (Supplementary Figure S3, *p* = 0.10).

### 3.2. Combined Annexin A2 and CA125 Has a High Sensitivity and Specificity in Diagnosing Stage I and Stage IA OC versus Healthy Controls

**Figure 2:**
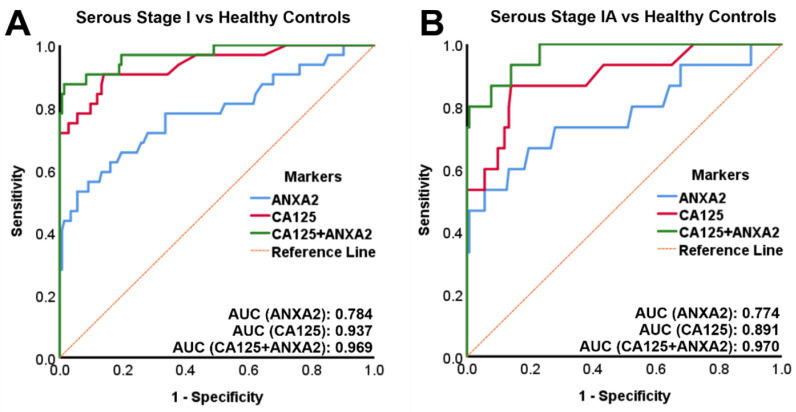
ROC curves for stage I OC versus healthy controls. (**A**) Plasma annexin A2 or CA125 alone and combined plasma annexin A2 + CA125 for stage I OC (*n* = 32) versus healthy controls (*n* = 143). (**B**) Plasma annexin A2 or CA125 alone and combined plasma annexin A2 + CA125 for stage IA OC (*n* = 15) versus healthy controls (*n* = 143).

The sensitivity and specificity in diagnosing early-stage OC versus healthy controls for either annexin A2 or CA125 alone and combined annexin A2 + CA125 was determined by logistic regression model and receiver operating characteristics (ROC) curve analysis. In stage I OC versus healthy controls, the area under the curve (AUC) for annexin A2 was 0.784 (40.6 sensitivity at 99.6 specificity) and for CA125 0.937 (71.9 sensitivity at 99.6 specificity). The AUC for combined annexin A2 + CA125 (0.969, 84.4 sensitivity at 99.6 specificity) was larger when compared to CA125 alone (Figure 2A + Table 1). In stage IA OC versus healthy controls, the AUC for annexin A2 was 0.774 (46.7 sensitivity at 99.6 specificity) and for CA125 0.891 (53.3 sensitivity at 99.6 specificity). The AUC for combined annexin A2 + CA125 (0.970, 80.0 sensitivity at 99.6 specificity) was larger when compared to CA125 alone (Figure 2B + Table 1). The accuracy in the diagnosis of stage IA OC versus healthy controls for annexin A2 (93.7%) or CA125 (95.6%) alone was increased by combining both markers (97.5% accuracy for annexin A2 + CA125) (Table 2).

### 3.3. Combined Annexin A2 and CA125 Has a High Accuracy in Diagnosing Stage IA OC versus Benign Ovarian Tumors

**Figure 3:**
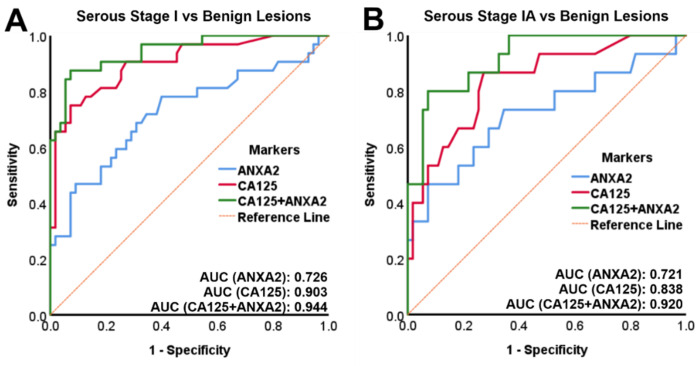
ROC curves for stage I OC versus benign ovarian tumors. (**A**) Plasma annexin A2 or CA125 alone and combined plasma annexin A2 + CA125 for stage I OC (*n* = 32) versus benign ovarian tumors (*n* = 55). (**B**) Plasma annexin A2 or CA125 alone and combined plasma annexin A2 + CA125 for stage IA OC (*n* = 15) versus benign ovarian tumors (*n* = 55). AUC: area under the curve; ROC: receiver operating characteristics.

The diagnostic accuracy in distinguishing early-stage OC from benign ovarian tumors was determined by logistic regression model and ROC curve analysis. In stage I OC versus benign ovarian tumors, the AUC for combined annexin A2 + CA125 was 0.944 and larger than for either annexin A2 (AUC = 0.726) or CA125 (AUC = 0.903) alone (Figure 3A). The AUC for combined annexin A2 + CA125 in stage IA OC versus benign ovarian tumors was 0.920 and also larger than for either annexin A2 (AUC = 0.721) or CA125 (AUC = 0.838) alone (Figure 3B). At 100% sensitivity, 45.5% specificity was achieved for combined annexin A2 + CA125 in stage I OC versus benign ovarian tumors, compared to either annexin A2 (3.6% specificity) or CA125 (20% specificity) alone. At 100% sensitivity, the specificity of diagnosing stage IA OC versus benign ovarian tumors was 63.6% compared to either annexin A2 (3.6% specificity) or CA125 (20% specificity) alone (Table 3). The diagnostic accuracy in distinguishing stage IA OC from benign ovarian tumors was almost twice as high for combined annexin A2 + CA125 (71.4%) in comparison to CA125 (37.1%) alone (Table 4).

## 4. Discussion

Ovarian cancer remains the most lethal gynecological cancer as it is frequently diagnosed at advanced stage. Early diagnosis and timely treatment are essential as mortality is closely related to stage of disease. The best strategy to improve OC survival would therefore be early detection through screening. However, an accurate early detection test does not exist.

CA125 remains the current gold standard as an OC biomarker. However, while CA125 is found to be elevated in about 80% of women with advanced disease, it is increased in less than 50% of stage I cases [18,19]. Furthermore, CA125 has a poor specificity as it is often elevated in benign ovarian tumors, resulting in false-positive results [6]. Consequently, CA125 alone is not a reliable and sensitive tumor marker for early OC diagnosis.

We recently modelled the metastatic microenvironment of OC in vitro and explored the two-way interactions between OC and peritoneal cells using proteomics [20]. A protein that was specifically modulated by this interaction was the phospholipid calcium binding protein annexin A2. Annexin A2 forms a complex with S100A10 and both together have a critical role in the plasminogen activator system which leads to the conversion of plasminogen to plasmin. Plasmin is a key enzyme which facilitates essential cellular processes involved in cancer invasion and metastasis [21]. We showed that annexin A2 is highly expressed in 90% of serous OC, is actively involved in the process of OC metastasis in vivo [16] and increased annexin A2 expression is associated with poor patient outcome [17]. To date, no reports have been presented on the potential diagnostic value of plasma annexin A2 in OC.

In the present study we demonstrate for the first time that plasma annexin A2 levels are significantly elevated in stage I, and in particular in stage IA OC patients, compared to healthy controls. However, logistic regression models followed by ROC curve analyses showed that plasma annexin A2 alone does not have sufficient accuracy for early diagnosis. The combination of plasma annexin A2 + CA125, however, had a sensitivity of 80% at 99.6% specificity in diagnosing stage 1A disease versus healthy controls. Similarly, annexin A2 together with CA125 achieved a sensitivity of 84.4% at 99.6% specificity in diagnosing stage I OC versus healthy controls.

As the prevalence of OC in the population is very low (1 in 2500 in postmenopausal women) and requirements for screening have to be very rigid to avoid potential morbidity from false-positive results, an effective OC screening test requires a minimum positive predictive value (PPV) of 10%. To achieve a PPV of 10%, a screening test needs to have a sensitivity of at least 75% and a specificity of at least 99.6% [4]. Our data, therefore, indicate that a combination of annexin A2 and CA125 would potentially fulfill these screening test requirements for OC.

Ovarian pathology is common, and it is estimated that 10% of all women undergo surgery for the investigation of an ovarian lesion during their lifetime. About 10% of ovarian masses are malignant in premenopausal women, compared to 20% in the postmenopausal group [22]. Predicting whether ovarian lesions are benign or malignant is important, as benign tumors might be managed conservatively but suspected malignant tumors have to be referred to gynecological oncology centers for potential radical surgery. As the timely management of OC significantly improves patient outcomes, tests are needed that permit accurate differential diagnosis

Our study reveals for the first time that the combination of annexin A2 + CA125 is able to distinguish early-stage IA OC from benign ovarian lesions (100% sensitivity, 63.6% specificity, 71.4% accuracy) more accurately than CA125 alone (100% sensitivity, 20% specificity, 37.1% accuracy). Therefore, the addition of annexin A2 to CA125 enables the detection of malignancies in patients with tumors that do not express CA125 and would therefore be missed by algorithms that employ CA125 alone.

Elevated circulating serum annexin A2 levels have been shown for malignancies of the liver [23,24,25], breast [26], lung [27], and stomach [28]. However, Gurluler et al. reported that serum annexin A2 was decreased in colorectal cancer patients compared to healthy controls [29]. In a large cohort study, serum annexin A2 was significantly elevated in early-stage hepatocellular carcinoma patients compared to healthy controls and patients with other malignancies such as lung or bowel cancer. The combination of annexin A2 and alpha-fetoprotein (AFP) improved the sensitivity in detecting early stage hepatocellular carcinoma [23]. Annexin A2 was also found to be significantly elevated in the plasma of breast cancer patients compared with healthy controls [30]. However, we did not observe a difference between plasma annexin A2 from patients with early-stage breast cancer compared to healthy controls. However, our breast cancer sample size was small, and the results need to be further validated in a larger cohort. Together, these reports indicate that annexin A2 is unlikely to be a specific OC biomarker.

We observed only a weak correlation between plasma annexin A2 levels and patient age. This observation confirms previous findings where annexin A2 levels were not associated with the age of patients in cancers of the liver [23,25], stomach [28] and bowel [29].

The role of circulating annexin A2 in early-stage OC progression remains unknown. Ulvestad et al. reported that annexin A2 is secreted into the serum by active secretion or shedding and not by proteolytic degradation [31]. Previous studies have shown the expression of annexin A2 in circulating tumor cells of breast cancer patients and suggested that secreted forms of annexin A2 might play a role in coagulation activation [32]. Annexin A2 containing extracellular vesicles were also found to be elevated in endometrial cancer patients compared to healthy controls [33].

We found higher annexin A2 levels in serum than in plasma EDTA. The reason for this difference is not clear but could be due to annexin A2 being released during blood clotting, as it plays a role in the vascular homeostasis and fibrinolysis [34].

Although our findings show that annexin A2 was elevated in advanced stage OC, we found that the combination of plasma annexin A2 and CA125 has a better diagnostic performance for early-stage OC in comparison to advanced cancer. The reason why annexin A2 is a better diagnostic marker for early-stage OC is unknown. Previous studies have reported annexin A2 cleavage by proteases, such as plasmin and matrix metallopeptidase 7 (MMP-7) [35,36]. Therefore, the secreted form of plasma annexin A2 could exist in different proteolytic forms in the early stage compared to advanced stage OC. This requires further investigation.

The strength of this study is the assessment of plasma annexin A2 levels in a large number of serous stage I (*n* = 32) and serous stage IA (*n* = 15) OC patients which is usually a limitation in OC biomarker studies. Furthermore, our samples were sourced from multiple international centers to avoid center-bias. However, our preliminary findings require further validation in a larger and independent cohort of early and advanced stage OC patients. This will also require the inclusion of other histological OC types, such as endometroid, clear cell and mucinous carcinomas.

## 5. Conclusions

In conclusion, plasma annexin A2 in combination with CA125 has potential as a diagnostic biomarker for the early detection of OC and shows significant diagnostic accuracy in predicting malignancy in women with ovarian lesions, warranting further investigations.

## Figures and Tables

**Table 1:** Sensitivity (%) of ANXA2, CA125 and ANXA2 + CA125 at 100%, 99.6% and 98% specificities, specificities (%) at fixed sensitivity (75%) and AUC from the ROC curve for stage I-IV OC versus healthy controls using the logistic regression model.

Marker	FIGO Stage	Sensitivity (100% Specificity)	Sensitivity (99.6% Specificity)	Sensitivity (98% Specificity)	Specificity (75% Sensitivity)	AUC
ANXA2	IA	33.3	46.7	46.7	72.0	0.774
	IA + IB	35.3	47.1	47.1	72.0	0.786
	IC	20.0	33.3	40.0	64.3	0.782
	I	28.1	40.6	43.8	66.4	0.784
	II	23.5	23.5	29.4	73.4	0.796
	III-IV	0	8.9	12.5	39.9	0.656
	I-IV	12.4	21.0	24.8	53.1	0.718
CA125	IA	53.3	53.3	53.3	88.1	0.891
	IA + IB	58.8	58.8	58.8	88.1	0.904
	IC	86.7	86.7	86.7	100	0.974
	I	71.9	71.9	71.9	97.2	0.937
	II	82.4	82.4	82.4	100	0.978
	III-IV	98.2	98.2	98.2	100	1.000
	I-IV	87.6	87.6	87.6	100	0.977
ANXA2+ CA125	IA	73.3	80.0	80.0	100	0.970
	IA + IB	70.6	82.4	82.4	99.3	0.973
	IC	86.7	93.3	93.3	100	0.975
	I	78.1	84.4	87.5	100	0.969
	II	82.4	82.4	82.4	100	0.975
	III-IV	98.2	98.2	100	100	1.000
	I-IV	87.6	89.5	92.4	100	0.986

**Table 2:** Accuracy (%) of ANXA2, CA125 and ANXA2 + CA125 calculated from the proportion of true positives, true negatives, false positives and false negatives in stage IA OC (*n* = 15) versus healthy controls (*n* = 143).

Marker	True Positive (TP)	True Negative (TN)	False Positive (FP)	False Negative (FN)	Accuracy (%)
ANXA2	5	143	0	10	93.7
CA125	8	143	0	7	95.6
ANXA2 + CA125	11	143	0	4	97.5

**Table 3:** Specificity (%) of ANXA2, CA125 and ANXA2 + CA125 at 100%, 94% and 75% sensitivity. AUC from the ROC curve for stage I-IV OC versus benign ovarian tumors using logistic regression model.

Marker	FIGO Stage	Specificity (100% Sensitivity)	Specificity (94% Sensitivity)	Specificity (75% Sensitivity)	AUC
ANXA2	IA	3.6	18.2	65.5	0.721
	IA + IB	3.6	18.2	65.5	0.736
	IC	5.5	7.3	60.0	0.714
	I	3.6	7.3	60.0	0.726
	II	5.5	25.5	67.3	0.733
	III-IV	3.6	5.5	36.4	0.573
	I-IV	3.6	5.5	50.9	0.646
CA125	IA	20.0	52.7	74.5	0.838
	IA + IB	20.0	52.7	74.5	0.856
	IC	54.5	92.7	98.2	0.956
	I	20.0	54.5	92.7	0.903
	II	72.7	74.5	98.2	0.951
	III-IV	92.7	94.5	98.2	0.991
	I-IV	20.0	74.5	98.2	0.958
ANXA2 + CA125	IA	63.6	67.3	94.5	0.920
	IA + IB	63.6	67.3	94.5	0.928
	IC	49.1	92.7	98.2	0.958
	I	45.5	67.3	94.5	0.944
	II	58.2	78.2	98.2	0.949
	III-IV	92.7	94.5	98.2	0.990
	I-IV	47.3	81.8	98.2	0.967

**Table 4:** Accuracy (%) of ANXA2, CA125 and ANXA2 + CA125 calculated from the proportion of true positives, true negatives, false positives and false negatives in stage IA OC (*n* = 15) versus benign ovarian tumors (*n* = 55).

Marker	True Positive (TP)	True Negative (TN)	False Positive (FP)	False Negative (FN)	Accuracy (%)
ANXA2	15	2	53	0	24.3
CA125	15	11	44	0	37.1
ANXA2 + CA125	15	35	20	0	71.4

## Data Availability

All available data is presented within the article or supplementary material.

## Supplementary Materials

The following are available online at https://www.mdpi.com/2075-4418/11/1/69/s1.

## Abbreviations

ANXA2	Annexin A2
AUC	Area under the curve
CA125	Cancer antigen 125
FIGO	International Federation of Gynecology and Obstetrics
HE4	Human epididymis protein 4
HGSOC	High grade serous ovarian cancer
OC	Ovarian cancer
PPV	Positive predictive value
ROC	Receiver operating characteristics
ROMA	Risk of ovarian malignancy algorithm
