# Supplementary material for: Diagnostic Value of Plasma Annexin A2 in Early-Stage High-Grade Serous Ovarian Cancer
Source: Diagnostics (Basel). 2021 Jan 4;11(1):69. doi: 10.3390/diagnostics11010069 (PMC7823988; doi:10.3390/diagnostics11010069)
Supplement: Supplementary file 1 [file diagnostics-11-00069-s001.pdf]

Supplementary materials

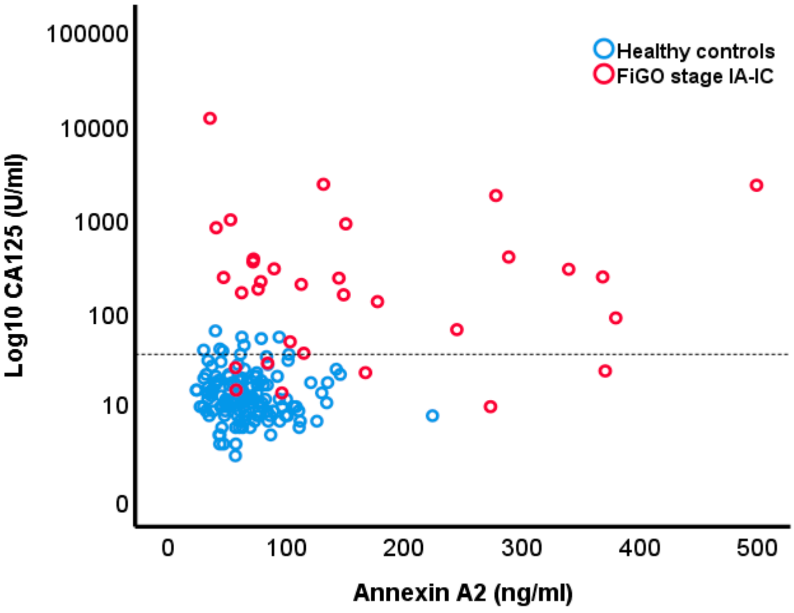

Supplementary Figure S1. Scatterplot for the correlation of plasma annexin A2 (ng/ml) and CA125 levels (U/ml) in OC (FIGO stage IA-IC, n=32, red) and healthy controls (n=143, blue). Dotted line represents CA125 cut-off value = 35U/ml. Stage I OC (n=7, red) with normal CA125 values (<35U/ml). Spearman's rho correlation (Correlation coefficient = 0.154, P=0.042).

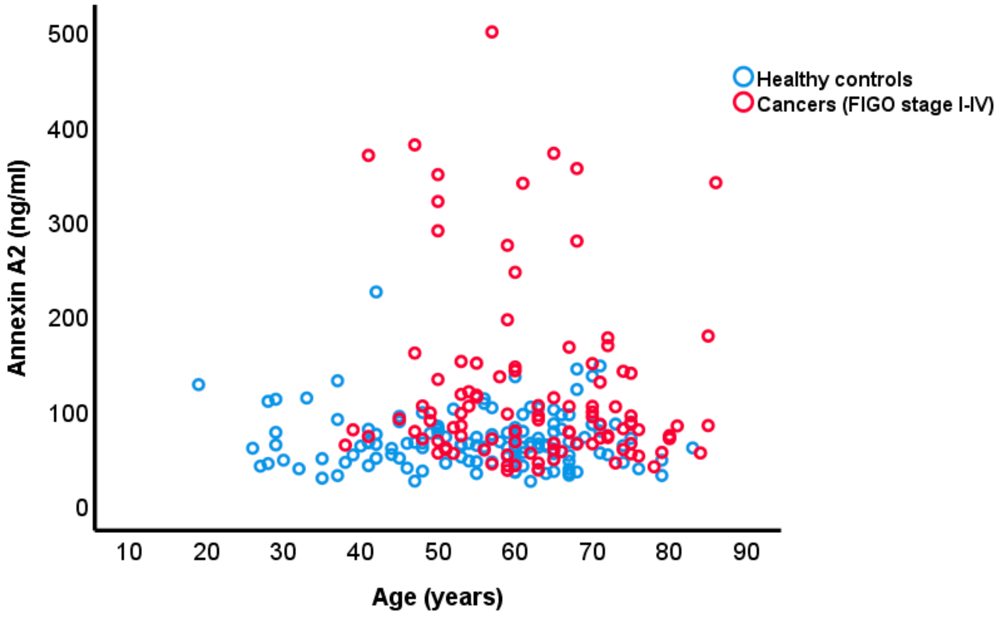

Supplementary Figure S2. Scatterplot for the correlation of plasma annexin A2 levels (ng/ml) and age (years) in OC (FIGO stage I-IV, n=105, red) and healthy controls (n=143, blue). Spearman's rho correlation (Correlation coefficient = 0.040, P=0.531).

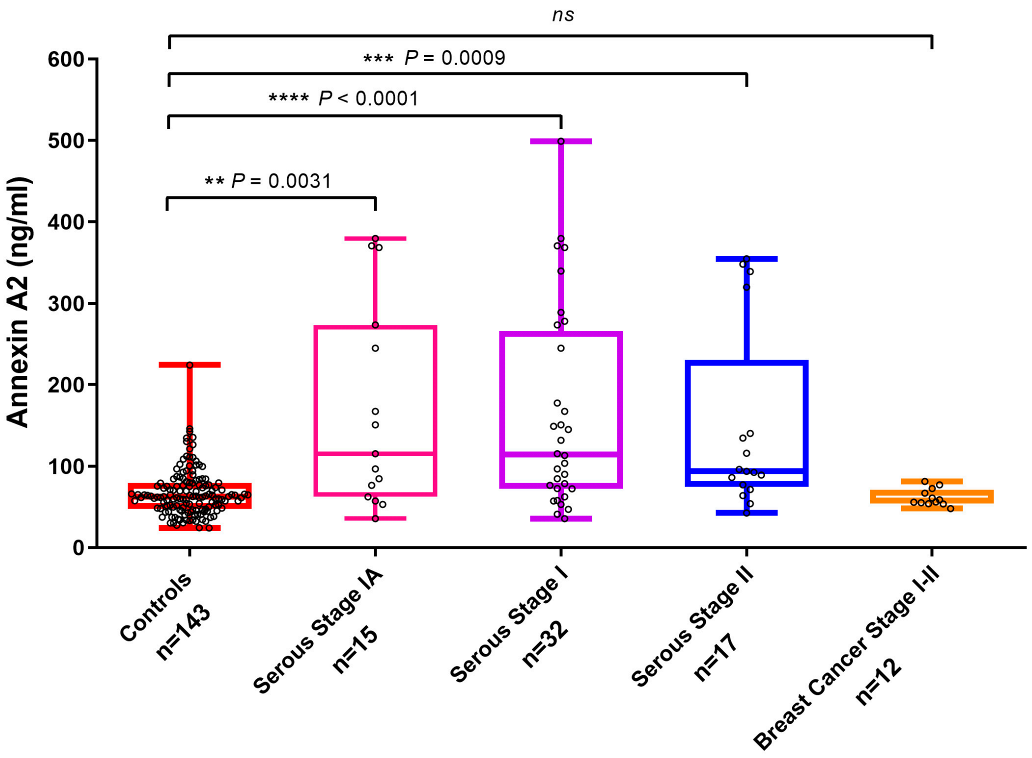

Supplementary Figure S3. Plasma annexin A2 levels in patients with breast cancer, serous ovarian cancer and healthy controls. Box and whisker plots representing plasma annexin A2 levels measured in stage IA (n=15), stage IA-IC (n=32) and stage II (n=17) OC, stage I-II (n=12) breast cancers and healthy controls (n=143). The median values for plasma annexin A2: Stage IA (115.3 ng/ml, range: 35.7 – 379.7), stage IA-IC (114.2 ng/ml, range: 35.7 – 499.1) and stage II (93.6 ng/ml, range: 42.6 – 354.7) serous ovarian cancers, stage I-II breast cancers (57.6 ng/ml, range: 48.1 – 81.4), and healthy controls (62.6 ng/ml, range: 24.2 – 224.3). The comparison between patient groups was performed by Kruskal-Wallis test and Dunn's multiple comparison test. (ns = not significant)

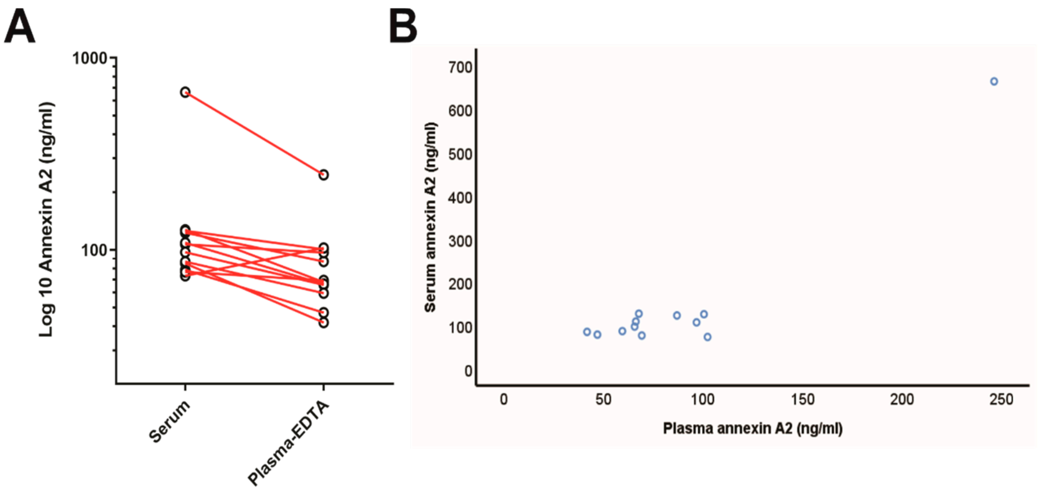

Supplementary Figure S4. (a) Serum annexin A2 and plasma annexin A2 levels in matching patient samples (n=12). (b) Scatterplot for the correlation of serum annexin A2 and plasma EDTA annexin A2 levels in matching patient samples (n=12). Spearman's rho correlation (Correlation coefficient = 0.364, P=0.245).

Supplementary Table S1. Clinicopathological characteristics of the patient cohort.

Patient Cohort	Disease Status	n	Age (Years) Median (range)	CA125 (U/ml) Median (range)
High grade serous ovarian cancers (n=105)	FIGO stage IA	15	59 (38-75)	154 (9-11710)
	FIGO stage IA-IC	32	58 (38-86)	206 (9-11710)
	FIGO stage IIA-IIC	17	60 (41-75)	298 (22-4710)
	FIGO stage IIIA-IIIC	54	67 (39-85)	903 (47-11200)
	FIGO stage IV	2	67 (63-70)	4760 (2050-7470)
Breast cancers (n=12)	Stage I	6	52 (41-66)	-
	Stage II	6	61 (46-68)	-
Benign ovarian lesions (n=55)	Ovarian serous cystadenoma	17	55 (28-87)	12 (3-320)
	Ovarian serous cystadenofibroma	9		
	Ovarian serous cyst	17		
	Ovarian fibroma	6		
	Ovarian endometrioma	1		
	Ovarian leiomyoma	1		
	Ovarian mucinous cystadenoma	1		
	Ovarian fibrothecoma	1		
	Mature cystic teratoma	1		
	Ovarian mesothelial inclusion cyst	1		
Healthy controls (n=143)	Healthy controls	143	60 (19-83)	12 (2-63)

Supplementary Table S2. (a) Patient numbers for each cohort from different centers and (b) median values for patient age and range (years).

Center	A. Total patient numbers								
	Controls	Benign	Stage IA	Stage IB	Stage IC	Stage I	Stage II	Stage III	Stage IV
**Royal Adelaide Hospital**	58	18	2	0	2	4	2	8	1
**Hudson Institute of Medical Research**	10	37	9	2	6	17	15	45	1
**Precision Med**	75	0	0	0	0	0	0	0	0
**Ontario Tumor Bank**	0	0	4	0	7	10	0	1	0
**Total**	143	55	15	2	15	31	17	54	2

Center	B. Patient Age (years), Median (range)								
	Controls	Benign	Stage IA	Stage IB	Stage IC	Stage I	Stage II	Stage III	Stage IV
**Royal Adelaide Hospital**	48 (19-79)	58 (28-83)	49 (38-59)	-	58 (50-65)	55 (38-65)	62 (60-63)	63 (50-85)	70
**Hudson Institute of Medical Research**	60 (53-74)	55 (39-87)	56 (41-72)	72 (68-76)	55 (51-86)	56 (41-86)	58 (41-75)	67 (39-84)	63
**Precision Med**	63 (30-83)	-	-	-	-	-	-	-	-
**Ontario Tumor Bank**	-	-	63 (55-75)	-	60 (45-85)	60 (45-85)	-	60	-
